# Assessing content validity of the functional assessment of cancer therapy–cutaneous T-cell lymphoma (FACT-CTCL): a qualitative study

**DOI:** 10.1186/s41687-026-01131-z

**Published:** 2026-06-23

**Authors:** Caroline Raymundo, Jenny Jung Sun Park, Mengyang Di, Cecilia Larocca, David Cella, Michi M Shinohara

**Affiliations:** 1 University of Washington School of Medicine, Seattle, USA; 2 Department of Dermatology, University of Washington, Seattle, USA; 3 Fred Hutchinson Cancer Center, Seattle, USA; 4 Department of Dermatology, Brigham and Women’s Hospital, Boston, USA; 5 Dana-Farber Cancer Institute, Boston, USA; 6 Department of Medical Social Sciences, Northwestern University Feinberg School of Medicine, Chicago, USA

**Keywords:** Cutaneous T-cell lymphoma, Mycosis fungoides, Sézary syndrome, Health-related quality of life, Patient-reported outcomes, Content validity, FACT-CTCL

## Abstract

**Background:**

Cutaneous T-cell lymphomas (CTCLs) are a heterogeneous group of non-Hodgkin lymphomas characterized by neoplastic T cells in the skin. Common subtypes of CTCL are mycosis fungoides (MF) and Sézary syndrome (SS). Patients with MF/SS can experience significant symptoms that affect health-related quality of life (HRQoL). Although the Functional Assessment of Cancer Therapy–Cutaneous T-Cell Lymphoma (FACT-CTCL) was developed as a disease-specific patient-reported outcome measure (PROM) for MF/SS, qualitative evaluation of its content validity in the target population has not been assessed. We therefore evaluated the content validity of the FACT-CTCL in patients with MF/SS and clinicians with expertise in MF/SS.

**Methods:**

Semi-structured cognitive interviews of patients with a diagnosis of MF or SS, as well as healthcare providers with expertise in treating MF/SS were conducted in alignment with the Consensus-based Standards for the Selection of Health Measurement Instruments (COSMIN). Patients evaluated the relevance, comprehensibility, and comprehensiveness of the FACT-CTCL. Clinicians evaluated relevance and comprehensiveness.

**Results:**

Twelve patients (9 MF, 3 SS) and eight clinician experts (3 oncologists, 4 dermatologists, 1 nurse practitioner) participated. The median age of patients was 68 years (range 36–77); six had early-stage (IA – IIA) disease and six had advanced-stage (IIB – IVB) disease. All items were deemed relevant by at least 95% of participants, and 44 of 47 items achieved 100% relevance across patients and clinicians. All items achieved at least 90% comprehensibility among patients, and 39 of 47 scored items were understood by all patients. All patients found the instructions and response options easy to understand, and all participants considered the instrument comprehensive.

**Conclusions:**

The FACT-CTCL demonstrated strong content validity in this sample of patients with MF/SS and expert clinicians. These findings support further evaluation and use of the FACT-CTCL as a disease-specific HRQoL instrument in MF/SS, while additional study in larger and more diverse populations remains warranted.

## Background

Cutaneous T-cell lymphomas (CTCLs) are heterogeneous non-Hodgkin lymphomas that primarily manifest in the skin. Mycosis fungoides (MF) and Sézary syndrome (SS) are two common subtypes of CTCL. Patients with MF/SS can experience a wide range of symptoms including severe pruritus, disfiguring skin lesions, infection, and psychological distress that can significantly affect physical, emotional, and social well-being [1–3].

The optimal instrument for assessing health-related quality of life (HRQoL) in patients with MF/SS remains unclear. A systematic review found substantial heterogeneity in the patient-reported outcome measures (PROMs) used in this population, with 30 different instruments identified across studies [4]. Among these, the Skindex-29 has been used most frequently [4–8]. However, as a skin-specific instrument, it does not fully address cancer-related psychosocial concerns that are highly relevant to many patients with MF/SS [1].

The Functional Assessment of Cancer Therapy–Cutaneous T-Cell Lymphoma (FACT-CTCL) is a disease-specific PROM developed to assess HRQoL in patients with MF/SS [9]. It combines the Functional Assessment of Cancer Therapy–General (FACT-G) with the Cutaneous T-Cell Lymphoma–Specific (CTCL-S), a 19-item subscale developed for patients with MF/SS. The FACT-CTCL has demonstrated favorable psychometric properties, but its content validity has not been assessed.

According to the Consensus-based Standards for the selection of health Measurement INstruments (COSMIN), content validity is the most important measurement property of a PROM and should be established before other measurement properties are interpreted [10, 11]. Content validity refers to the extent to which an instrument adequately reflects the construct of interest in the target population and includes relevance, comprehensiveness, and comprehensibility [10]. Input from both patients and relevant clinicians is important to determine whether instrument content appropriately captures the intended construct within its clinical context. Notably, content validity as defined by COSMIN has not been established for PROMs used in studies of patients with MF/SS [4]. We therefore conducted a qualitative study to evaluate the relevance, comprehensiveness, and comprehensibility of the FACT-CTCL in patients with MF/SS and clinicians with expertise in MF/SS.

## Methods

In this qualitative study, semi-structured interviews were performed following guidelines from the COSMIN manual [11]. Data reporting is per the Standards for Reporting Qualitative Research [12]. This study received Institutional Review Board approval by the University of Washington (STUDY00013375).

### FACT-CTCL instrument

The FACT-CTCL [9] is a disease-specific patient-reported outcome measure used to assess HRQoL in patients with MF/SS. The instrument incorporates the FACT-G, a 27-item questionnaire developed to assess HRQoL in patients with cancer across four domains: physical, social, emotional, and functional well-being. A non-scored instruction item preceding the sexual well-being question, which allows respondents to opt out of answering this item, was also included in the content validity assessment. The FACT-CTCL additionally includes a 19-item CTCL–S subscale, which was developed through a multistep process involving qualitative interviews with 18 MF/SS patients and 8 expert clinicians, item prioritization, and expert-guided selection. All scored items are rated on a 5-point Likert scale (0–4) reflecting experiences over the prior 7 days and are reverse coded per FACIT scoring guidelines such that higher scores indicate better HRQoL.

### Recruitment

Adult patients (≥ 18 years old) with a confirmed diagnosis of MF or SS were recruited from a single academic institution (University of Washington/Fred Hutchinson Cancer Center). Healthcare providers with at least 5 years of experience in treating patients with MF or SS and/or membership in the following cutaneous lymphoma organizations: United States Cutaneous Lymphoma Consortium (USCLC), International Society for Cutaneous Lymphomas (ISCL) and/or Cutaneous Lymphoma International Nursing Network (CLINN), or International Dermatology Outcome Measures (IDEOM) CTCL workgroup were recruited through professional contacts. To be eligible for the study, patients and providers needed to be able to read and speak English fluently and have access to telephone or a computer that allowed video chat. Patient participants were assigned a unique identification (ID) number to ensure confidentiality. The recruitment goal was set to a minimum of 10 patients and a minimum of 7 healthcare providers per the COSMIN guidelines [10].

### Semi-structured cognitive interviews: overview

All interviews were conducted by a trained interviewer following an interview guide developed by the research team. Development of the interview guide was informed by COSMIN guidelines for evaluation of content validity studies of patient-reported outcome measures (PROMs) [10, 11]. Interviews were conducted over telephone or secure video conference. All participants provided verbal consent for the researchers to record audio, video, and a transcript of their cognitive interview. Interviews were transcribed verbatim.

### Patients

Patients were first invited to informally describe their experience with MF/SS in their own words as a cognitive warm-up. This was intended to encourage patients to provide reflections on their diagnosis, symptoms, experience with treatment, and how their condition has affected their quality of life and to build rapport between the interviewer and participant.

Patients were then asked to evaluate the relevance and comprehensibility of the instructions and each sequential item in the FACT-CTCL to their personal experience with MF/SS, as well as the overall comprehensiveness of the instrument. Patients were asked to ‘think-aloud’ as they reviewed the instrument instructions and completed items of the FACT-CTCL [13]. The interviewer did not provide any aid to the patients as they completed the questionnaire. Follow-up or “verbal probing” questions were asked by the interviewer to further evaluate the patient’s understanding [14]. Examples of verbal probing questions include asking the patient to verbalize any ambiguities in the wording of the items or asking the patient to describe what experiences related to their condition were prompted after reading an item.

Patient demographics such as race/ethnicity and gender identity were collected directly from the patient at the time of the interview. Age and clinical characteristics such as years since diagnosis with MF/SS, CTCL subtype, and disease stage were extracted from the electronic medical record by the study team.

### Clinician experts

Similar to the patient interviews, healthcare providers with expertise in treating MF/SS were asked to evaluate each item of the FACT-CTCL, including the instructions and recall time, and its relevance to their patient population with MF/SS. Healthcare providers were asked to evaluate the comprehensiveness of the FACT-CTCL and were provided with the opportunity to describe any important HRQoL concepts that they felt were missing from the FACT-CTCL.

Healthcare provider demographics and clinical practice characteristics such as race/ethnicity, gender, medical specialty, years since residency or fellowship, practice setting, participation in CTCL research, and average weekly MF/SS caseload were gathered directly from the healthcare provider.

### Data analysis

Audio/video recordings were transcribed verbatim. Data were extracted from the transcripts with a focus on the evaluation of relevance, comprehensibility, and comprehensiveness in the patient interviews, and relevance and comprehensiveness in the clinician interviews, guided by the ten criteria for good content validity described by COSMIN [10]. A cutoff of > 85% in all three categories (relevance, comprehensibility, and comprehensiveness) was used to determine good evidence for content validity [10]. Thematic content analysis was used to analyze the qualitative interview data by coding the verbatim interview transcripts and grouping codes into thematic categories.

## Results

### Participant demographics

Twelve patients and eight healthcare providers were recruited and included; patient characteristics are summarized in Table 1. Among patients, 8/12 (66.7%) were male and 11/12 (91.7%) were white. The median age was 68 years. Nine (75.0%) patients had MF, and three (25.0%) had SS. There was an equal distribution of early-stage (IA – IIA) and advanced-stage (IIB – IVB) disease.

**Table 1:** Patient demographic and clinical characteristics (*n* = 12)

Characteristic	*N*(%) unless otherwise indicated
Sex	
Female	4 (33.3%)
Male	8 (66.7%)
Age, years	
Mean	64.7
Median (range)	68 (36–77)
Race	
White	11 (91.7%)
Asian	1 (8.3%)
CTCL subtype	
MF	9 (75%)
SS	3 (25%)
Disease stage	
Early (IA-IIA)	6 (50%)
Advanced (IIB-IVB)	6 (50%)

Characteristics of the eight healthcare providers enrolled are summarized in Table 2. A majority of the healthcare providers were female (7/8, 87.5%). Half of the participants were white and half were Asian. Two-thirds of providers (5/8, 62.5%) saw more than 10 MF/SS patients weekly in clinical practice on average.

**Table 2:** Healthcare provider demographics and clinical practice characteristics (*n* = 8)

Characteristics	*N*(%)
Gender	
Female	7 (87.5%)
Male	1 (12.5%)
Race	
White	4 (50%)
Asian	4 (50%)
Medical specialty	
Dermatology	3 (37.5%)
Hematology/oncology	4 (50%)
Oncology nursing	1 (12.5%)
Years since last residency or fellowship	
1–5	2 (25%)
6–10	2 (25%)
11–15	1 (12.5%)
>15	3 (37.5%)
Average number of cutaneous T-cell lymphoma patients seen in clinical practice weekly	
1–2	0 (0%)
3–10	3 (37.5%)
>10	5 (62.5%)

### Overall relevance and comprehensibility

All patients (12/12, 100%) stated that the instrument instructions and response options were easy to understand. All patients and 7/8 (87.5%) of healthcare providers found the one-week recall period relevant to their condition or their patients. One healthcare provider stated that the one-week recall period is relevant to early-stage patients, but may be too short for some patients with advanced-stage disease because of the waxing and waning nature of HRQoL.

### Item-level relevance

All scored items in the FACT-CTCL were deemed relevant by at least 95.0% of patient and provider participants, and 44 of 47 scored items were considered relevant by 100% of patient and provider participants (Tables 3 and 4). The three items without 100% consensus were GF1 (‘I am able to work’), GF2 (‘My work is fulfilling’), and CTCL 4 (‘My skin feels irritated’). One patient stated that items GF1 and GF2 were not relevant because they were currently retired. When asked to describe their thoughts on item CTCL 4, a patient stated, “I mean, a person can be irritated, but skin can’t be irritated. I guess it means a combination of itching, raw, and painful. Maybe.”

**Table 3:** Functional Assessment of Cancer Therapy – General (FACT-G) item tracking matrix

FACT-G Item	Patient and provider relevance (*n = 20*)	Patient comprehensibility *(n = 12)*
GP1. I have a lack of energy	20/20 (100%)	11/12 (91.7%)
GP2. I have nausea	20/20 (100%)	11/12 (91.7%)
GP3. Because of my physical condition I have trouble meeting the needs of my family	20/20 (100%)	12/12 (100%)
GP4. I have pain	20/20 (100%)	12/12 (100%)
GP5. I am bothered by side effects of treatment	20/20 (100%)	12/12 (100%)
GP6. I feel ill	20/20 (100%)	12/12 (100%)
GP7. I am forced to spend time in bed	20/20 (100%)	12/12 (100%)
GS1. I feel close to my friends	20/20 (100%)	12/12 (100%)
GS2. I get emotional support from my family	20/20 (100%)	12/12 (100%)
GS3. I get support from my friends	20/20 (100%)	12/12 (100%)
GS4. My family has accepted my illness	20/20 (100%)	12/12 (100%)
GS5. I am satisfied with family communication about my illness	20/20 (100%)	12/12 (100%)
GS6. I feel close to my partner (or the person who is my main support)	20/20 (100%)	12/12 (100%)
Q1. Regardless of your current level of sexual activity, please answer the following question. If you prefer not to answer it, please check this box, and go to the next section	18/18 (100%)*	9/10 (90%)*
GS7. I am satisfied with my sex life	18/18 (100%)*	9/10 (90%)*
GE1. I feel sad	20/20 (100%)	11/12 (91.7%)
GE2. I am satisfied with how I am coping with my illness	20/20 (100%)	12/12 (100%)
GE3. I am losing hope in the fight against my illness	20/20 (100%)	12/12 (100%)
GE4. I feel nervous	20/20 (100%)	11/12 (91.7%)
GE5. I worry about dying	20/20 (100%)	12/12 (100%)
GE6. I worry that my condition will get worse	20/20 (100%)	12/12 (100%)
GF1. I am able to work (include work at home)	19/20 (95.0%)	12/12 (100%)
GF2. My work (include work at home) is fulfilling	19/20 (95.0%)	12/12 (100%)
GF3. I am able to enjoy life	20/20 (100%)	12/12 (100%)
GF4. I have accepted my illness	20/20 (100%)	12/12 (100%)
GF5. I am sleeping well	20/20 (100%)	12/12 (100%)
GF6. I am enjoying the things I usually do for fun	20/20 (100%)	12/12 (100%)
GF7. I am content with the quality of my life right now	20/20 (100%)	12/12 (100%)

*Two patients elected to skip this item

**Table 4:** Cutaneous T-Cell Lymphoma – Specific (CTCL-S) item tracking matrix

CTCL-S Item	Patient and provider relevance (*n = 20*)	Patient comprehensibility (*n = 12)*
CTCL 1. My skin itches	20/20 (100%)	12/12 (100%)
CTCL 2. My skin feels sensitive or “raw”	20/20 (100%)	12/12 (100%)
CTCL 3. I have painful cracks in the skin on my hands or feet	20/20 (100%)	12/12 (100%)
CTCL 4. My skin feels irritated	19/20 (95.0%)	11/12 (91.7%)
CTCL 5. I worry that my skin condition may get worse	20/20 (100%)	12/12 (100%)
CTCL 6. My skin bleeds easily	20/20 (100%)	12/12 (100%)
CTCL 7. I am embarrassed by the appearance of my skin	20/20 (100%)	12/12 (100%)
CTCL 8. I am frustrated by my skin condition	20/20 (100%)	12/12 (100%)
CTCL 9. My skin is dry or “flaky”	20/20 (100%)	12/12 (100%)
CTCL 10. I have trouble falling asleep because of my skin condition	20/20 (100%)	12/12 (100%)
CTCL 11. My family is bothered by my skin condition	20/20 (100%)	12/12 (100%)
CTCL 12. I have difficulty doing things with my hands because of the condition of my skin	20/20 (100%)	12/12 (100%)
CTCL 13. I have difficulty walking because of my skin condition	20/20 (100%)	11/12 (91.7%)
CTCL 14. I am bothered by the red or pink appearance of my skin	20/20 (100%)	12/12 (100%)
CTCL 15. I am bothered by the uncertainty of my prognosis	20/20 (100%)	12/12 (100%)
CTCL 16. My skin condition interferes with my social life	20/20 (100%)	12/12 (100%)
CTCL 17. I am tired of dealing with my skin condition	20/20 (100%)	12/12 (100%)
CTCL 18. I have painful lesions or tumors on my skin	20/20 (100%)	12/12 (100%)
CTCL 19. I am bothered by the inconvenience of treatment for my skin condition	20/20 (100%)	12/12 (100%)

### Item-level comprehensibility

All items in the FACT-CTCL were felt to be easily comprehensible by at least 90.0% of patients, while 39 of 47 items were considered easily comprehensible by 100% of patients (Tables 3 and 4). Items with less than 100% comprehensibility included: GP1 (‘I have a lack of energy’), GP2 (‘I have nausea’), Q1 (item on preference to answering GS7), GS7 (‘I am satisfied with my sex life’), GE1 (‘I feel sad’), GE4 (‘I feel nervous’), CTCL 4 (‘My skin feels irritated’), and CTCL 13 (‘I have difficulty walking because of my skin condition’).

One patient stated that items GP1 (‘I have a lack of energy’) and GP2 (‘I have nausea’) were difficult to answer because they were unsure whether to respond based on fatigue or nausea as a consequence of their disease or as a side effect of treatment. The patient further elaborated that they had not experienced fatigue or nausea as a symptom of their disease, stating, “Yesterday, I had lack of energy, but yesterday I got the infusion… So, I think, you know, overall, I don’t feel like I have a lack of energy, you know, from the disease.”

Similar to items GP1 and GP2, one patient found items GE1 (‘I feel sad’) and GE4 (‘I feel nervous’) difficult to understand because the items do not specify sadness or nervousness related to their disease or due to any cause. When the patient was evaluating items GE1 and GE4, they stated, “These statements [GE1 and GE4] are so vague… Does this mean, I feel bad because of my illness, or are you asking me if I feel sad in general?”

Items Q1 (item on preference to answering GS7) and GS7 (‘I am satisfied with my sex life’) were confusing to one patient. When responding to these items, the patient stated “I guess the only confusion, it would be hard to answer these questions if you weren’t sexually active, right? Regardless of your current level of activity… Yeah I think the more I read it, the more confused I get by it.”

One patient felt item CTCL 13 (‘I have difficulty walking due to my skin condition’) was difficult to answer because they were unclear whether the question was addressing difficulty walking due to disease-associated joint or muscular pathology, or whether the item was asking the respondent to think about how skin-specific symptoms like irritation affect their ability to walk. The patient stated, “I wasn’t really sure what this means. Well, I mean now it’s 0, but you know, even in my active state, when I was bothered by it, I mean, the clothes irritating my skin was there, but I wouldn’t relate that as to difficulty walking.”

### Comprehensiveness

100% of patients and healthcare providers agreed that the FACT-CTCL is comprehensive and covers all important or required aspects of HRQoL in CTCL. One healthcare provider noted that there are no items in the FACT-CTCL that assess the impact of skin discoloration on quality of life. Although the provider felt that this is an important concept to assess, they did not feel it is required, and thus agreed that the FACT-CTCL is comprehensive.

## Discussion

In this qualitative evaluation of the FACT-CTCL, participants generally judged the instrument to be relevant, understandable, and comprehensive for assessing HRQoL in MF/SS. A small number of items prompted minor ambiguity, particularly when patients were asked to distinguish disease-related experiences from treatment effects or more general emotional states. Overall, these findings suggest that the FACT-CTCL captures many of the domains important to patients with MF/SS while also identifying a few areas where item interpretation may vary.

These findings should be interpreted within the evolving MF/SS PROM landscape. A systematic review of 103 MF/SS publications from 2000 to 2022 found substantial heterogeneity in the HRQoL PROMs used and did not identify any content validity study meeting COSMIN criteria for PROMs in this population [4]. A review of lymphoma PROMs publications from 2015 to 2025 included four CTCL trials, and similarly highlighted the ongoing need for robust, disease-specific HRQoL measurement in this population and noted limitations of existing instruments in comprehensively capturing domains important to patients with CTCL [15]. Prior qualitative work in CTCL likewise found that many patient concerns, including itch, sleep disturbance, appearance-related concerns, uncertainty, treatment burden, and skin-specific symptoms such as flaking and skin breaks, were not adequately or consistently represented in existing QoL measures [16]. Several of these concepts are represented in the FACT-CTCL, and in the present study all patient and clinician participants considered the instrument comprehensive. These findings support the relevance of further qualitative evaluation of disease-specific HRQoL instruments in MF/SS.

Several disease-specific HRQoL instruments have emerged for MF/SS, reflecting increasing recognition that generic and skin-specific measures may not fully capture the patient experience. The MF/SS-CTCL QoL [17] was developed using patient concept elicitation and cognitive debriefing in an MF/SS population, providing some evidence relevant to content validity, although a COSMIN-style content validity evaluation has not been reported. More recently, preliminary validation of the CTCL-PRO-18, another emerging CTCL-specific instrument was described [18]. Together with the FACT-CTCL, these efforts support an evolving measurement landscape in MF/SS. Within the COSMIN framework, content validity is considered the most important measurement property, and judgments about whether a PROM is relevant, comprehensive, and comprehensible rely on evidence from patients and relevant experts [10, 11]. Accordingly, transparently reported qualitative studies add important evidence for evaluating disease-specific HRQoL instruments in this population.

Strengths of our study include adherence to COSMIN’s guidelines for good content validity methodology [11], including recommendations for cognitive interview methodology, volume of patient and provider participation, and qualitative analysis. Additional strengths include representation of both early-stage (IA–IIA) and advanced-stage (IIB–IVB) disease and inclusion of clinicians from multiple relevant specialties.

Limitations of our study include the small sample size and limited representation of patients with SS. Given the more aggressive and clinically distinct nature of SS, transferability of these findings to patients with SS should be interpreted cautiously, and additional targeted content validation in larger SS cohorts is warranted. There was also a relatively narrow distribution of patient demographic characteristics in our study, particularly with respect to race and age. The incidence of MF/SS is significantly higher in Black patients compared with white patients, with a relative risk ratio of 1.56 when adjusted for age and household income [19]. In our study, white patients were overrepresented, and no Black/African American participants were included. The average age of our participants was 65 years, which is higher than the average age of 57 years at diagnosis for patients with MF [20]. Patients were also recruited from a single academic center, and only English-speaking participants were eligible, which may further limit transferability.

## Conclusions

The FACT-CTCL demonstrated strong content validity in this sample of patients with MF/SS and clinicians with expertise in MF/SS. The instrument showed high relevance and comprehensibility, and participants did not identify major gaps in overall comprehensiveness. These findings provide important qualitative evidence supporting the FACT-CTCL as a disease-specific HRQoL instrument for MF/SS, while additional validation in larger and more diverse populations, particularly among patients with SS, remains needed.

## Data Availability

The data underlying this article cannot be shared publicly for the privacy of individuals that participated in the study. The data will be shared on reasonable request to the corresponding author.

## Abbreviations

CLINN: Cutaneous Lymphoma International Nursing Network
COSMIN: Consensus–based Standards for the selection of Health Measurement Instruments
CTCL: Cutaneous T–cell lymphoma
CTCL-S: Cutaneous T–Cell Lymphoma–Specific subscale
FACIT: Functional Assessment of Chronic Illness Therapy
FACT-CTCL: Functional Assessment of Cancer Therapy–Cutaneous T–Cell Lymphoma
FACT-G: Functional Assessment of Cancer Therapy–General
HRQoL: Health–related quality of life
IDEOM: International Dermatology Outcome Measures
IRB: Institutional Review Board
ISCL: International Society for Cutaneous Lymphomas
MF: Mycosis fungoides
PRO: Patient–reported outcome
PROM: Patient–reported outcome measure
SS: Sézary syndrome
USCLC: United States Cutaneous Lymphoma Consortium
